# Supplementary material for: The RNA-binding protein ZC3H11A interacts with the nuclear poly(A)-binding protein PABPN1 and alters polyadenylation of viral transcripts
Source: J Biol Chem. 2023 Jun 23;299(8):104959. doi: 10.1016/j.jbc.2023.104959 (PMC10371797; doi:10.1016/j.jbc.2023.104959)
Supplement: Supporting Figures S1–S4 and Table S1 [file mmc1.pdf]

The RNA-binding protein ZC3H11A interacts with the nuclear poly(A)-binding protein PABPN1 and alters polyadenylation of viral transcripts

Katharina Kases, Erik Schubert, Zamaneh Hajikhezri, Mårten Larsson, Priya Devi, Mahmoud Darweesh, Leif Andersson, Göran Akusjärvi, Tanel Punga, Shady Younis

Figure S1. A comparison between human and mouse ZC3H11A interactome.

Figure S2. MS analysis of ZC3H11A-Flag interacting proteins in HeLa cells.

Figure S3. Immunofluorescence analysis of the ZC3H11A-Flag protein in nuclear speckles in HeLa cells.

Figure S4. Expression levels of the ZC3H11A mutant proteins in HAdV-5-infected A549 cells.

Table S1. List of PCR primers used in this study.

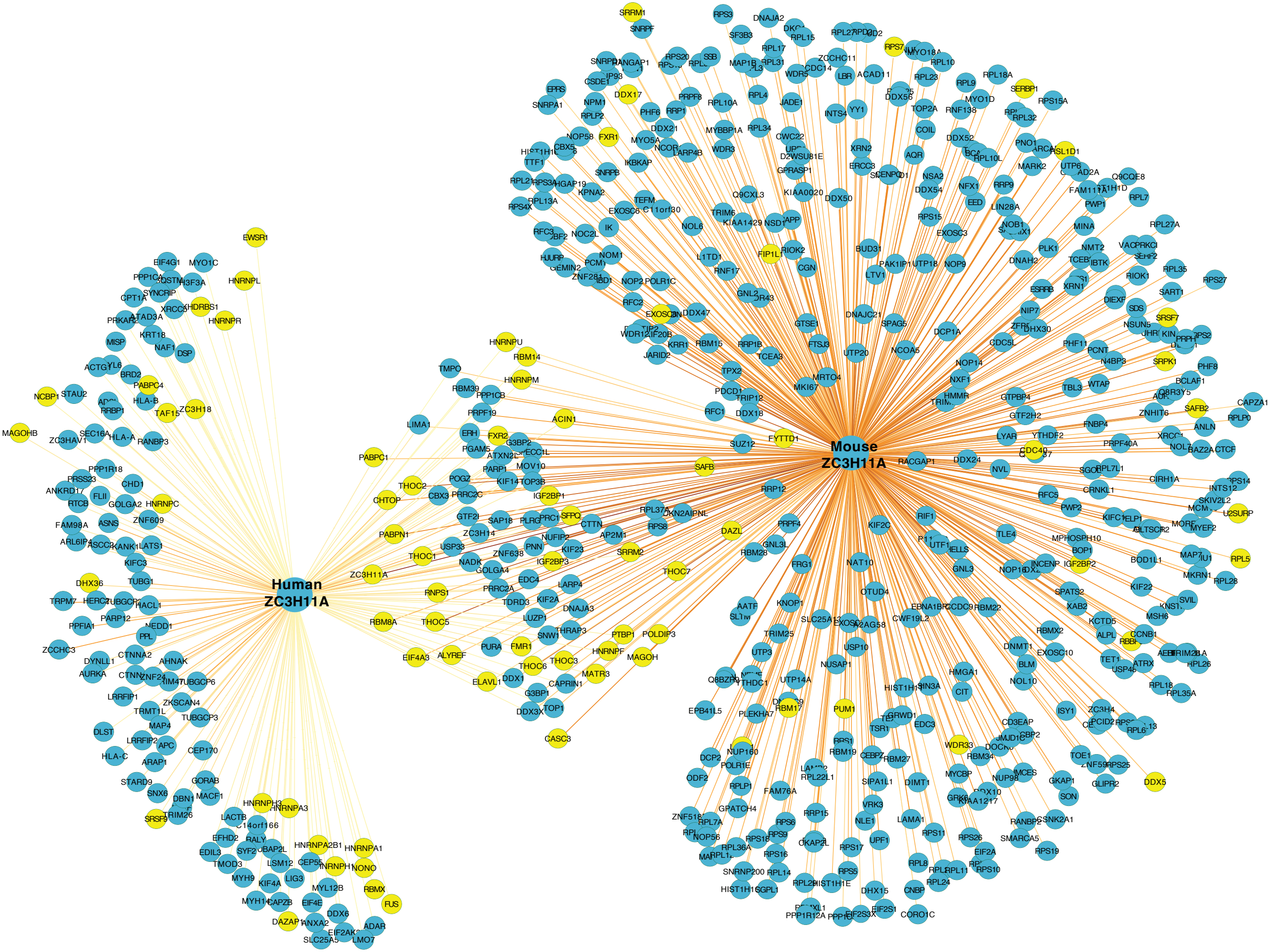

Figure S1. A comparison between human and mouse ZC3H11A interactome. Network analysis of the identified ZC3H11A-interacting partners ZC3H11A in human (HeLa) and mouse (mESCs) cells based on co-Ips (adjusted P <0.05). Yellow circles highlight proteins involved in 3'UTR mRNA processing based on the STRING database.

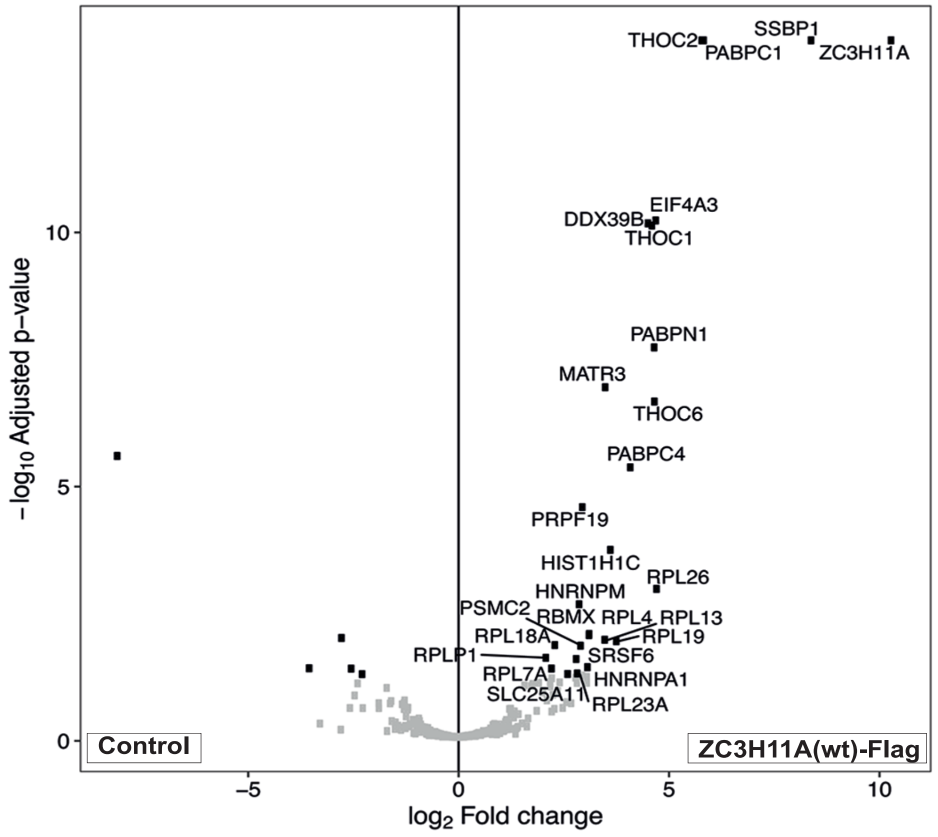

Figure S2. The ZC3H11A-Flag interacts with subunits of the TREX complex and PABPs. Volcano plot showing identified proteins that specifically interact with the ZC3H11A(wt)-Flag protein in HeLa cells. An adjusted P-value cut off of 0.05 and a log2 fold change cut off of 2 was used. Data are shown from a biological triplicate experiment. Control; cells transfected with pcDNA(C-Flag) plasmid, ZC3H11A(wt)-Flag; cells transfected with pcDNA-ZC3H11A(wt)-Flag plasmid.

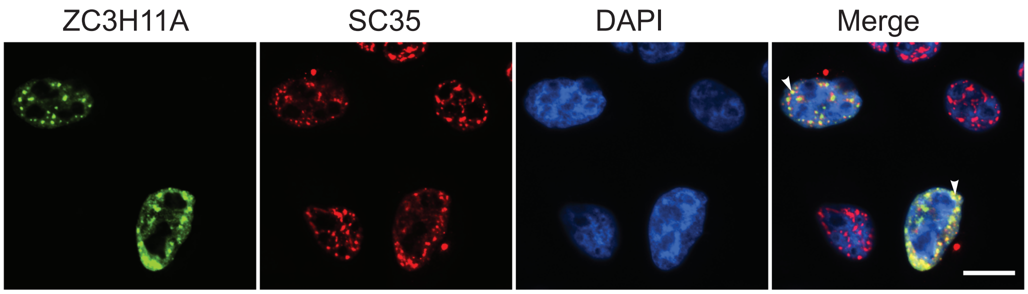

Figure S3. ZC3H11A localization into nuclear speckles. HeLa cells were transfected with the ZC3H11A-Flag encoding plasmids for 24 h. Indirect immunofluorescence (IF) was done with the anti-Flag and anti-SC35 antibodies. Cells were counterstained with DAPI. White arrowheads indicate typical colocalization of the SC35 and ZC3H11A-Flag. Scalebar is 20 µm.

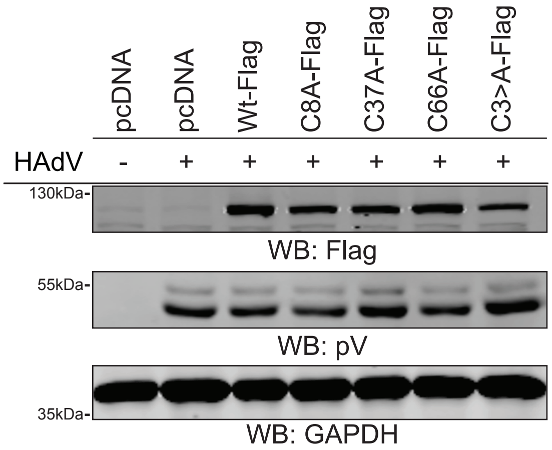

Figure S4. Expression levels of the ZC3H11A-Flag mutant proteins in HAdV-5-infected A549 cells. Cells were transfected with respective plasmids for 24h, followed by HAdV-5 infection (MOI= 5, 48h). Whole cell lysates were separted on 9% SDS-PAGE and western blot was done using the anti-Flag (detects ZC3-Flag), anti-pV (virus capsid protein, marker for HAdV-5 infection) and anti-GAPDH antibodies.

Table S1.

List of PCR primers used in this study

**Target**<br>Purpose	**Forward primer (5’-3’)**	**Reverse primer (5’-3’)**
**SNHG10**<br>qRT-PCR	GTTATTGACTTCCTACCCAGCA	CTGGAATCAATGAATCACGTTC
**SNHG19**<br>qRT-PCR	CGTCCAGGCCTGGCCTAC	GCTCGCGACGAAACCTGC
**HAdV-5 pVI**<br>qRT-PCR	AGGCGTCTAACCAGTCACAGTC	CTTGCCAGTTTCCCATGAACGG
**HAdV-5 pVII**<br>qRT-PCR		GCCCCAGCCTGTGTTATTGC
**HAdV-5 Hexon**<br>qRT-PCR	CGAGGGACCTGAGCGAGTC	TGTAAGACCACTGCGGCATC
**HAdV-5 Fiber**<br>qRT-PCR		GAGGACCGGTTTCCGTGTCA
**HPRT1**<br>qRT-PCR	TGGACAGGACTGAACGTCTTG	CCAGCAGGTCAGCAAAGAATTTA
**ePAT**<br>Jänicke., 2012		GCGAGCTCCGCGGCCGCGTTTTTTTTTTTT
**TVN-PAT**<br>Jänicke., 2012		GCGAGCTCCGCGGCCGCGTTTTTTTTTTTTVN
**ePAT/TVN-PAT Universal**<br>Jänicke., 2012		GCGAGCTCCGCGGCCGCGT
**ePAT/TVN-PAT**<br>HAdV-5 L3 pA	GCAGCCACAGTGCGCAGATTAGGAG	
**ePAT/TVN-PAT**<br>HAdV-5 L5 pA	CCATTACACTAAACGGTACACAGGA	
